# Suitability of electrolyzed oxidizing water for the disinfection of hard surfaces and equipment in radiology

**DOI:** 10.1186/s40201-015-0160-8

**Published:** 2015-01-28

**Authors:** Robert Pintaric, Joze Matela, Stefan Pintaric

**Affiliations:** Department of Radiology, University Medical Centre Maribor, Maribor, Slovenia; University of Ljubljana, Veterinary faculty, Ljubljana, Slovenia

**Keywords:** Biocide, Electrolyzed oxidizing water (EOW), Disinfection, Micro-organisms

## Abstract

**Background:**

Hospitals are faced with increasingly resistant strains of micro-organisms. When it comes to disinfection, individual parts of electronic equipment of angiology diagnostics such as patient couches of computer tomography (CT) and magnetic resonance imaging (MRI) scanners prove to be very hard to disinfect. Disinfectants of choice are therefore expected to possess properties such as rapid, residue-free action without any damaging effect on the sensitive electronic equipment. This paper discusses the use of the neutral electrolyzed oxidizing water (EOW) as a biocide for the disinfection of diagnostic rooms and equipment.

**Methods:**

The CT and MRI rooms were aerosolized with EOW using aerosolization device. The presence of micro-organisms before and after the aerosolization was recorded with the help of sedimentation and cyclone air sampling. Total body count (TBC) was evaluated in absolute and log values.

**Results:**

The number of micro-organisms in hospital rooms was low as expected. Nevertheless, a possible TBC reduction between 78.99–92.50% or 50.50–70.60% in log values was recorded.

**Conclusions:**

The research has shown that the use of EOW for the air and hard surface disinfection can considerably reduce the presence of micro-organisms and consequently the possibility of hospital infections. It has also demonstrated that the sedimentation procedure is insufficient for the TBC determination. The use of Biocide aerosolization proved to be efficient and safe in all applied ways. Also, no eventual damage to exposed devices or staff was recorded.

## Background

Modern radiological imaging examinations are expected to provide detailed, swift and reliable examination yielding maximum diagnostic data while representing minimum burden on the patient. This is the reason why more and more electronic equipment is built into the diagnostic devices to acquire maximum information on patient’s condition in the shortest time possible. Also, with the ever-growing number of diagnostic examinations, the possibility of hard surface contamination with micro-organisms from infected patients is increasing as well. Such surfaces may represent a possible source of infections for other patients and medical staff [1-6]. One of the key factors of a successful disinfection procedure is disinfectant’s exposure time. The selection of a disinfectant, however, is not an easy task to do. The basic requirement is rapid, residue-free action. Today, there are altogether 250 substances that are known to have biocidal effect. According to the World Organisation for Animal Health (OIE), a total of 154 are used independently or in combination with other biocides. The highlighted problem becomes so much greater once we take into account the increase of the hospital infections (such as MRSA for instance) and the growing number of patients infected with the infectious disease causing agents [7-16]. Bearing in mind the increasing number of electronic equipment built into diagnostic devices, residue-free surfaces are of crucial importance. The more equipment the greater the possibility of corrosive actions causing damage to the device’s vital parts, especially when we take into account that the biocides themselves have a corrosive effect. Touch screen command modules of the state-of-the-art angiographic diagnostics, for instance, are extremely sensitive to various cleaning agents and disinfectants containing alcohol and other substances with corrosive effect. Accessibility for cleaning represents an additional problem. Patient couches of computer tomography (CT) and magnetic resonance imaging (MRI) scanners are very hard to access when it comes to cleaning and disinfection [17-19]. Consequently, new approaches to disinfection procedures have been studied. Thanks to its mechanism of action, neutral electrolyzed oxidizing water (EOW) has been considered as a possible biocide of the new generation [20,21]. The principle of the EOW production has been known for some time now. Basically, the alkaline ionized water and acid oxidized ionized water are generated from diluted non-iodised cooking salt (NaCl solution), whereby the alkaline fraction reaches a pH of 11–12, while the acid one has a pH of 1–3. While the alkaline ionized water is considered to have a cleaning effect, the acid one has extremely biocidal effect. Mostly, the effect of the EOW action has been attributed to the pH change only. However, more detailed analysis has revealed that electro-oxidized water works through several mechanisms. Among them, the most important ones for the biocidal effect are the redox potential, generated oxidized and super-oxidized ions, and to a smaller extent the produced chlorides, sodium hypochlorite and residual chlorine. Moreover, EOW is characterized by a marked deficiency of electrons due to which it has a tendency for electro-neutral environment that can be achieved only through the abstraction of electrons from the surrounding environment. If there are any micro-organisms in that environment, EOW abstracts the electrons from their membrane disrupting their balance and thereby causing their death. The production of oxidized and super-oxidized ions classifies EOW among the biocidal agents that give off oxygen. An important characteristic of this group of agents is rapid action and ecologically acceptable breakdown products. From the point of view of weaknesses, the most important ones are violent reaction with any existing organic substances, special storage requirements (special containers with air-vent valves that relieve excess pressure in the container), corossivity and respiratory system irritation as well as causticity. The above-mentioned substances are produced during the electrolysis and are classified among biocidal substances. They are produced in very small quantities. One of the key factors in disinfection is acidity, whereby a pH between 1–3 is very acidic and has biocidal effect as such. Not to forget, however, that low pH values expose surfaces to corrosive acting [22-28]. Thanks to its mechanism of action, EOW has a broad spectrum of activity. It has proved itself as a very good agent in the presence of biofilms. The main substance of the pH is supposed to be hypochioric acid which is in nature produced by white blood cells in their fight against pathogenic microbes (attacking the microbe cell membrane by dissolving protective membrane’s biofilm) [29-33]. Numerous articles list wide EOW application possibilities in the fields, for instance plant production [34], pig production (preventive disinfection), poultry and food processing industries [35]. It has shown particularly good results in cold fogging of the rooms and can also be listed in the disinfection program in the event of any epizootic disease outbreak. In humane medicine, it is applied both for the disinfection of the surgical instruments such as endoscopes as well as for the hospital hard surface cleaning.

## Methods

In its search for a possible solution, the Radiology Department of the Maribor University Medical Centre (UKC) decided to test the air samples and the samples taken from the test surfaces of the various types of diagnostic equipment in order to establish the actual condition and the scope of the micro-organism presence in the air and their significance for the surface contamination. Ethical Commission of the Republic of Slovenia approved the study under the serial number 110/05/11. Additionally, the possibility of the air aerosolizationwith the EOW was tested. More specifically, the product tested was EOV Steriplant® N produced by OBISAN –Institute for Biotechnological Research and Development from Murska Sobota, Slovenia. The available commercial form of the product contains sodium hypochlorite, chlorate, chlorine dioxide and ozone. Its pH value ranges between 6 and 8 and its redox potential is +800 ± 100 mV. The research involved 6 diagnostic pieces of equipment, thereof two for angiology, two for CT and two for MRI respectively. The purpose of the research was to establish the efficacy of the applied EOW biocidal action on the present bioaerosol. The identification of the micro-organism presence in the air and on surfaces was carried out to establish the level of the contamination in order to be able to determine the importance of the reduction of the micro-organisms present in the air with the EOW aerosolisation. To determine the micro-organism presence, the micro-organism sedimentation method was applied directly on the surface of the prepared medium. The medium was placed horizontally on the surface and fixed vertically. Following the expiry of the one hour exposure time, the plates with the collected micro-organisms were taken to the laboratory. The second air sample collection method involved Coriolis Air Sampler (produced by Coriolis, France) using cyclone technology. Through the whirling motion of the medium and with the help of the centrifugal force, the samples were collected to a bioaerosol and prepared for further treatment. With the air flow rate of 300 liters per minute, altogether 1,200 litres of air were pulled through the liquid collection media during the collection time of 4 minutes. As a liquid medium, the sterile physiological saline was used in which the bioaerosol from the air was collected. All collected samples from the 6 diagnostic rooms were taken while the air ventilation system was off and they were transported to laboratory for further treatment at the temperature of 4°C. Plates with medium collected according to the sedimentation method were incubated for 48 hours. Following the expiry of that time, the grown colonies were counted (ISO 132697/2002). Air samples collected in the suspension of the physiological saline were first diluted and then sown to a medium. Depending on the cultures grown, further determination was carried out (ISO 4833/2003). What followed was the counting of micro-organisms and the determination of their actual total number.

## Results and discussion

**Figure 1:**
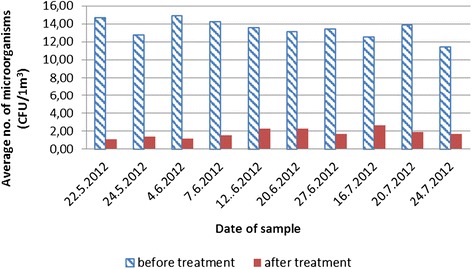
**Total number of micro** **-** **organisms and the presence of micro** **-** **organisms after the EOW aerosolization** **(absolute values)** **.**

**Figure 2:**
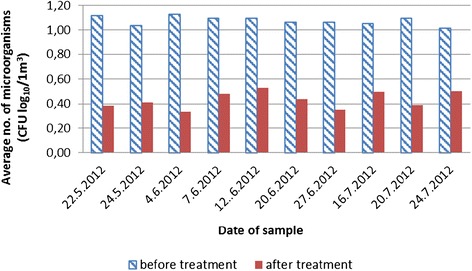
**Total number of micro-** **organisms and the presence of micro-** **organisms after the EOW aerosolization** **(calculated to log**_**10**_ **).**

The research involved 6 diagnostic rooms, 600 air samples collected in the liquid medium and 50 samplings on exposed mediums. The values of the samples gathered are shown in the tables below. For the sake of greater clarity, they are displayed as graphs. As anticipated, the number of micro-organisms present in the diagnostic room air was low. Clearly, to get a more reliable confirmation of the decrease in the number of micro-organisms it is preferable – from the point of view of the aerosol biocide action efficiency – to ensure as high initial number of micro-organisms as possible. However, this research was determining the reliability of action in actual conditions. As a result, the recorded decreases were smaller than they might have been in experimental conditions. On average, the total average number of micro-organisms in all rooms was between 11.43– 14.87 CFU/m^3^ of the sampled air (Figure 1). Expressed in log values it totaled 1.01–1.13 CFU log_10_/m^3^ (Figure 2).

**Figure 3:**
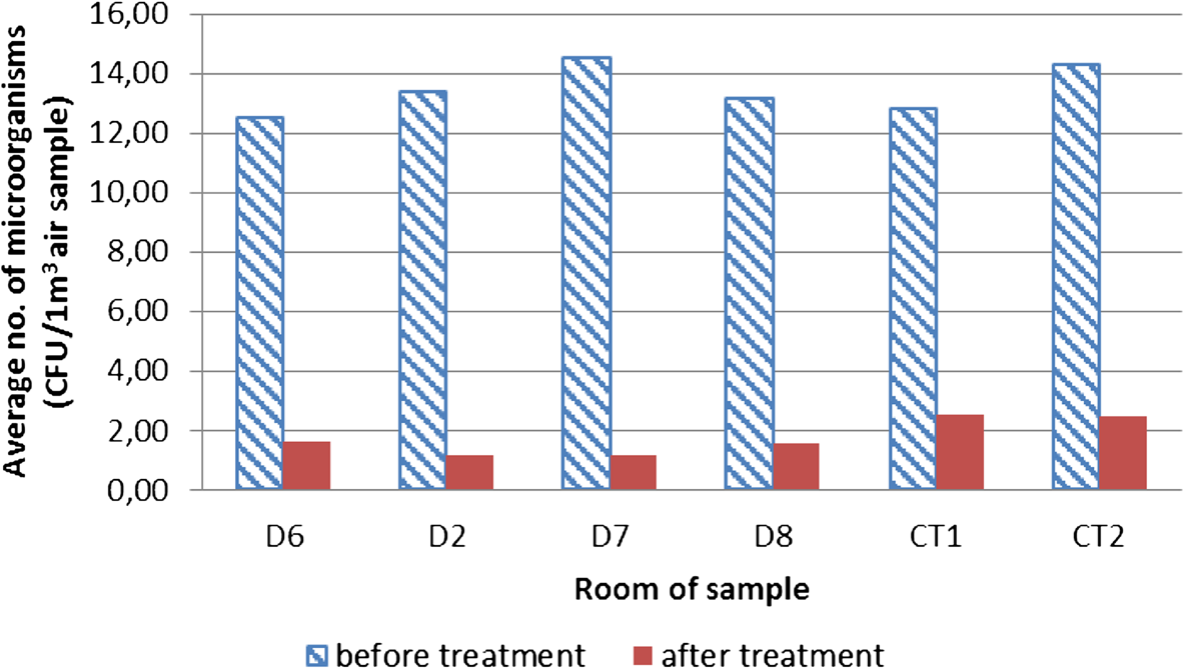
**Total number of micro-organisms in diagnostic rooms (absolute values).**

**Figure 4:**
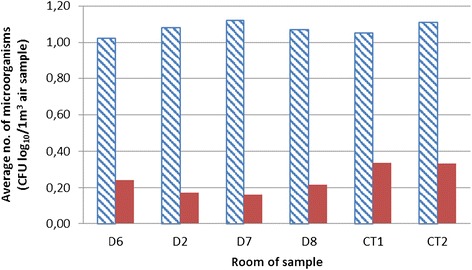
**Total number of micro-organisms in diagnostic room (calculated on log**_**10**_ **).**

During the air sampling, the two most affected rooms by micro-organisms were the diagnostic room D7 (on average 14.50 CFU/m^3^) and CT2 (on average 14.32 CFU/m^3^) (Figure 3), relative to log values of 1.11 for D7 and 1.12 CFU log10/m^3^ for the room CT 2 (Figure 4).

**Figure 5:**
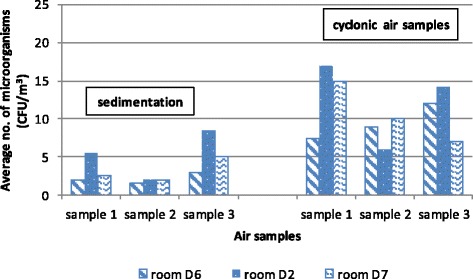
**The total number of micro-organisms determined by the sedimentation procedure and cyclone method-based sampling in diagnostic rooms.**

Figures 1 and 2 display a relatively small presence of micro-organisms during the sampling period. The main reason for that is controlled diagnostic room ventilation through air conditioning systems. A comparison of data for all rooms together before and after the EOW aerosolization shows a 78.99–92.50% decrease in the total number of micro-organisms (Figure 1). Log comparisons display a reduction between 0.71-0.96 log_10_ CFU/m^3^ (Figure 2). A comparison of individual room data before and after the EOW aerosolization shows 80.19–92.14% decrease of the total micro-organism number (Figure 3). Log comparisons display a decrease between 0.51-0.80 log_10_ CFU/m^3^ (Figure 4). The presence of micro-organisms was also determined with the help of the sedimentation procedure on Petri’s plates with medium. The results were compared with the cyclone sampling method. It was found that the sedimentation procedure detected 28.57–82.67% less micro-organisms in the air compared to cyclone sampling method as shown in Figure 5.

During the counting the forms of colonies were observed as well. Any suspicious colonies were subject to further determination. However, in none of the cases studied there were no particularly dangerous agents to health.

## Conclusions

The results of air sampling in the microbiological burden of the diagnostic rooms showed a fairly uniform load of microorganisms. We believe that the reason of an identified microbial presence in the way of forced air ventilation spaces of the entire hospital (central ventilation system with air prior preparation). Particularly worrisome is the presence of microorganisms in the operating rooms intended for surgery because of possible postoperative complications. Recent studies by other authors highlight the importance of microbial aerosolization in the operating rooms [36] and the importance of burden rooms with micro-organisms for the presence of microorganisms in the respiratory system of health personnel [37]. Recommendations of some authors are that the diagnostic and operational spaces achieve the presence of microorganisms of less than 10 CFU/m^3^ airs [38,39]. According to the results of our study it was for 25-45% microorganisms higher than recommended. Biocide aerosol-based on EOW showed a reduction in CFU in the air spaces of test on average values from 1.14 to 2.54 CFU/m^3^ or decrease of 80.19 to 92.14%. According to the recommendations of the authors [38,39] we believe that the use of the biocide aerosol Steriplant® N in practical terms in prepared space in which substantially reduce the burden of microorganisms. We believe that this helps to establish a bio-security between operational and diagnostic interventions. Considering the fact that the biocide aerosolization need 6–8 ml of biocide solution/1 m^3^ of air can reach very small amounts of disinfectant effects in operation rooms and equipment. Important features of the biocide Steriplant ®N hospital environment is a broad spectrum of activity mainly in the form of resistant microorganisms (metycillin resist with S. aureus, E. coli) uncorrosivity, security for operators disinfection, medical staff and patients, and that does not remain on the surfaces of the biocide residues (not required disposal of residues). We also wish to highlight the importance of the choice of the methodology air sampling for the presence of microorganisms. We believe that the compulsory cyclone method of air sampling in the liquid medium is appropriate to identify the presence of micro-organisms in relation to the sedimentation process, as we found from 28.57 to 82.67% for the higher levels of microorganisms.
